# The Aggregated Electromagnetic Vortex Wave and Multi-Modal Imaging Experiment

**DOI:** 10.3390/s25216578

**Published:** 2025-10-25

**Authors:** Caipin Li, Xiaomin Tan, Shitao Zhu, Shengyuan Li, Dong You, Jiao Liu, Wencan Peng, Tao Wu, Yifeng He, Kang Liu, Zhuo Zhang

**Affiliations:** 1 Xi’an Institute of Space Radio Technology, Xi’an 710100, China; licaipin2010@163.com (C.L.); lishengyuan05@163.com (S.L.); youdong@cast504.com (D.Y.); liujiao202509@163.com (J.L.); wcanpeng1@163.com (W.P.); wut1@cast504.com (T.W.); yiifenghe@cast504.com (Y.H.); 2 School of Information and Communications Engineering, Xi’an Jiaotong University, Xi’an 710049, China; shitaozhu@xjtu.edu.cn; 3 School of Electronic Science, National University of Defense Technology, Changsha 410073, China; lk2018@ustc.edu.cn; 4 Aerospace Information Innovation Research Institute, Chinese Academy of Sciences, Beijing 100101, China; zhangzhuo@aircas.ac.cn

**Keywords:** electromagnetic vortex waves, multi-modal imaging, vortex wave generation method

## Abstract

Electromagnetic vortex waves have received widespread attention in many fields due to their unique physical characteristics. The information dimension provided by vortex electromagnetic waves brings possibilities for future breakthroughs in radar detection and imaging. This article proposes a multi-modal aggregated electromagnetic vortex wave generation method for the first time. Moreover, it conducts vehicle imaging experiments to verify the method’s practicality. The core element of the experiment is to simultaneously generate multiple-mode electromagnetic vortex wave signals with energy accumulation and perform fusion processing. Firstly, multiple orbital angular momentum (OAM) modes are superimposed to generate a mode group, and the initial phase of the modes in the mode group is further controlled to synthesize aggregated electromagnetic vortex waves. Based on the generation of aggregated vortex waves, imaging experiments were conducted using a vehicle-mounted setup. The experimental procedure and multi-modal fusion results were presented. It has been shown that the energy of the main lobe signal of the image target is enhanced by utilizing multi-modal vortex radar information fusion, which can improve the signal-to-noise ratio of the target imaging.

## 1. Introduction

Radar has been widely used in various industries due to its ability to work in all weather conditions. However, with the deepening of applications, the requirements for obtaining target information are becoming increasingly stringent. Electromagnetic vortex wave radar, as a new type of radar system, has received widespread attention since its concept was proposed. The orbital angular momentum-provided new information dimension enables acquiring more target-related information. Compared with traditional radar, multi-modal electromagnetic vortex waves have important application advantages in super-resolution imaging and rotational Doppler extraction [1].

Many research institutions have conducted research on electromagnetic vortex waves. In [2], vortex electromagnetic waves were generated through a sparse circular phased array. Guan et al. [3] generated multi-modal OAM waves using polarization-controlled metasurface antennas. Orfeo et al. [4] developed a phased array electromagnetic vortex wave ground-penetrating radar. Yin et al. [5] designed a network structure based on a diode-adjustable phase, achieving ±1, ±2, and ±3 OAM modes. Li et al. [6] proposed a vortex array design method based on curve fitting and demonstrated through microwave anechoic chamber measurements that the antenna significantly improved the energy aggregation of multi-modal OAM waves. Zhang et al. [7] generated large-scale OAM modes by sacrificing time resources using a rotating antenna system. In 2021, Yang et al. [8] generated electromagnetic vortex waves with a mode of 2 using a single feed circular patch antenna; in the same year, Shahmirzadi et al. [9] generated electromagnetic vortex waves using a sparse circular phased array; Yang et al. [10] generated electromagnetic vortex waves using a chiral-triggered rotation-selectable metasurface antenna. In 2022, electromagnetic vortex waves were generated by chiral-triggered rotatable metasurface antennas [11]. In 2023, Zhang et al. [12] designed a single-layer uniform circular array antenna, which generated a conical OAM beam by geometric rotation of the single-layer patch. Reference [13] designed an X-band multimode antenna and verified the super-resolution imaging performance of electromagnetic vortex radar in the microwave anechoic chamber. Reference [14] combined electromagnetic vortex wave radar with SAR imaging, but this experimental mode only had one mode of information. In 2025, Zhen et al. [15] found that in the field of optics, in addition to scalar vortex waves, there are also vector vortex waves. Similarly, electromagnetic vortex waves also exist in vector and scalar forms. In addition, the electromagnetic vortex wave beam mentioned above has a circular intensity profile and energy voids in the propagation direction [16,17,18,19]. As the propagation distance increases, the energy void continues to expand. There are significant limitations in application, especially for radar detection, where energy is crucial [20,21,22,23,24].

This article utilizes multiple modes of aggregate electromagnetic vortex wave information to conduct field experiments for the first time and integrates multiple modes of information for imaging processing. The relevant research results demonstrate that the imaging fusion of multiple modes of information can improve the signal-to-noise ratio of the target.

## 2. Conventional Electromagnetic Vortex Wave Generation

Assuming that the signal emitted by the electromagnetic vortex radar is a linear frequency modulation signal, the emission waves of different modes can be as follows:

(1) $$S\left(t,l\right)=rect\left(t/T\right)\times \exp\left\{j\pi Kt^{2}\right\}\times \exp\left\{j\pi f_{c}t\right\}\times \exp\left\{jl_{n}\phi_{n}\right\}$$

In the equation, t is the time variable, T is the pulse duration period, K is the frequency modulation rate of the linear frequency modulation signal, and $f_{c}$ is the center frequency of the signal. $\phi_{n}$ represents the azimuth position angle of the antenna unit, and $l_{n}$ represents the nth angular momentum mode.

After multi-modal phase integration of the echo signal, the above equation can be expressed as follows:

(2) $$\begin{array}{c} S_{e}\left(t,l\right)\approx rect\left[\left(t-t^{\prime }\right)/T\right]\times \exp\left\{j\pi K{\left(t-t^{\prime }\right)}^{2}\right\}\times \exp\left\{j2\pi f_{c}\left(t-t^{\prime }\right)\right\}\times \sum_{n=0}^{N-1}\exp\left\{jxa\sin\theta \cos\left(\phi -\phi_{n}\right)\right\}\times \exp\left\{jl_{n}\phi_{n}\right\}\\ \;\;\;\approx N\times \exp\left\{jl_{n}\pi /2\right\}\times \exp\left\{jl_{n}\phi \right\}\times rect\left[\left(t-t^{\prime }\right)/T\right]\times \exp\left\{j\pi K{\left(t-t^{\prime }\right)}^{2}\right\}\times \exp\left\{j2\pi f_{c}\left(t-t^{\prime }\right)\right\}J_{1}\left(xa\sin\theta \right) \end{array}$$

In the formula $t^{\prime }=2R/c$, *R* is the operating distance from the vortex radar to the target point, ϕ represents the azimuth position angle between the antenna and the target, $x=2\pi \left[K\left(t-t^{\prime }\right)+f_{c}\right]/c$, *c* is the speed of light, a is the array radius, and θ is the pitch angle position of the target relative to the axis of the vortex electromagnetic wave beam, $i^{\prime }=e^{jl\pi /2}$.

After the fusion of electromagnetic vortex waves of different modes, the signal expression can be rewritten as follows:

(3) $$S_{e}\left(t,l\right)=\sum_{n=1}^{\infty }S_{e}\left(t,l_{n}\right)=\sum_{n=1}^{\infty }N\times \exp\left\{jl_{n}\pi /2\right\}\times \exp\left\{jl_{n}\phi \right\}\times rect\left[\left(t-t^{\prime }\right)/T\right]\times \exp\left\{j\pi K{\left(t-t^{\prime }\right)}^{2}\right\}\times \exp\left\{j2\pi f_{c}\left(t-t^{\prime }\right)\right\}J_{1}\left(xa\sin\theta \right)$$

where $l_{n}$ represents the nth angular momentum mode.

In order to conduct multi-modal imaging fusion experiments, electromagnetic vortex radar equipment was developed. The electromagnetic vortex antenna, used in the experiment under a concentric ring array, can generate modal information simultaneously. The antenna array elements are optimized to ensure that each loop of the array elements and the number of vortex wave modes meet the following relationship:

(4) $$N\geq 2{\left|l_{n}\right|}_{\max}$$

## 3. Generation of Aggregated Electromagnetic Vortex Waves

Traditional OAM beams have a circular intensity profile and energy holes [25,26,27,28,29,30,31,32,33,34]. Owing to the inherent divergence characteristic of these beams, as the propagation distance increases, the energy holes keep expanding. When electromagnetic vortex waves of various modes maintain consistent divergence angles and propagation directions, beam-forming via electromagnetic wave eigenmode superposition can be achieved by reasonably avoiding phase singularity in the propagation path. Multiple specific OAM modes are selected and superimposed to form a mode group (MG); then, the intensity and initial phase of each mode in the MG are further adjusted to synthesize the aggregated beam. The synthesized beam has azimuthal beam diversity, which has the potential to construct aggregated electromagnetic waves.

### 3.1. The Theory of Aggregated Electromagnetic Vortex Waves

Because the energy of OAM beams converges in the radial direction and exhibits a uniform intensity distribution in the azimuth direction, the OAM of order $l_{n}$ can be represented as follows:

(5) $$E_{n}^{PSOAM}=A_{n}e^{j(l_{n}\varphi +\varphi_{n})}$$

(6) $$S_{e}\left(t,l\right)\approx N\times \exp\left\{il\pi /2\right\}\times \exp\left\{il\phi \right\}\times rect\left[\left(t-t^{\prime }\right)/T\right]\times \exp\left\{i\pi K{\left(t-t^{\prime }\right)}^{2}\right\}\times \exp\left\{i2\pi f_{c}\left(t-t^{\prime }\right)\right\}J_{1}\left(xa\sin\theta \right)$$

Here, it is assumed that a mode group is composed of *N* OAM modes with OAM orders of $l_{1}$, $l_{1}+\Delta l$, …., $l_{1}+(N-1)\Delta l$, denoted as the modular group MG {$l_{1}$, $l_{1}+\Delta l$, …., $l_{1}+(N-1)\Delta l$}. The antenna pattern function of the shaped beam in terms of angle can be expressed as follows:

(7) $$F\left(\varphi \right)=\sum_{n=1}^{N}A_{n}e^{j(l_{n}\varphi +\varphi_{n})}=\sum_{n=1}^{N}A_{n}e^{j\left\{\left[l_{1}+(n-1)\Delta l\right]\varphi +\varphi_{n}\right\}}$$

In the above equation, $A_{n}$ and $\varphi_{n}$ represent the strength and initial phase of the mode group, respectively, $l_{1}$ represents the initial mode in the mode group, Δl represents the mode interval, and *N* represents the total number of OAM modes in the mode group. In the case of equal amplitude and phase, assuming that the intensity of each mode in the mode group is one and the initial phase is set to 0°, the antenna pattern function can be rewritten as follows:

(8) $$F\left(\varphi \right)=\sum_{n=1}^{N}A_{n}e^{j(l_{n}\varphi +\varphi_{n})}=\sum_{n=1}^{N}A_{n}e^{j\left\{\left[l_{1}+(n-1)\Delta l\right]\varphi +\varphi_{n}\right\}}$$

According to Euler’s formula, the antenna pattern function of the mode group can be further rewritten as follows:

(9) $$F\left(\varphi \right)=e^{jl_{1}\varphi }\cdot \frac{e^{j\frac{N\Delta l}{2}\varphi }}{e^{j\frac{\Delta l}{2}\varphi }}\cdot \frac{\sin\left(\frac{N\Delta l\varphi }{2}\right)}{\sin\left(\frac{\Delta l\varphi }{2}\right)}=\frac{\sin\left(\frac{N\Delta l\varphi }{2}\right)}{\sin\left(\frac{\Delta l\varphi }{2}\right)}\cdot e^{j(l_{1}+\frac{N-1}{2}\Delta l)\varphi }$$

In the above equation, $l_{1}$ represents the initial mode in the mode group, Δl represents the mode interval, *N* represents the total number of OAM modes in the mode group, and φ represents a phase of the mode group.

From the above equation, it can be seen that the antenna pattern function of the electromagnetic vortex wave can be divided into the product of the amplitude term and the phase term. The amplitude term describes the beam intensity distribution of the mode group, which is related to the total number of modes *N* and the mode interval but independent of the initial mode. The phase term describes the phase distribution of the mode group in the azimuth direction, which is related to *N*, ∆l, and the initial mode.

**Figure 1:**
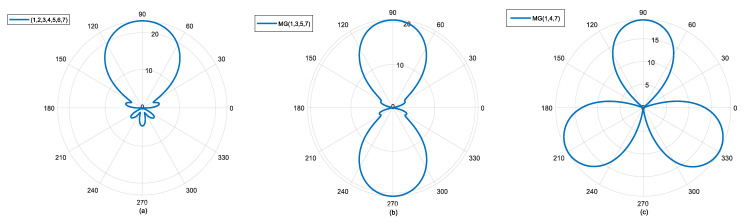
Multi-beam generation using a beam-forming method based on OAM mode group. (**a**) 1 mode interval; (**b**) 2 mode interval; (**c**) 3 mode interval.

To illustrate the influence of modal spacing on beam shape, we assume three scenarios: modal groups MG {1, 2, 3, 4, 5, 6, 7}, MG {1, 3, 5, 7}, and MG {1, 4, 7}, with modal spacing of 1, 2, and 3 for each modal group. Their initial modal settings remain consistent. The simulation results are shown in the following Figure 1.

From Figure 1, it can be seen that the number of main lobes is determined, corresponding to one main lobe, two main lobes, and so on. If you want to obtain more main lobes, a larger modal interval needs to be adopted when constructing the modal group. For imaging purposes, only one main lobe is required, so the modal spacing is usually set to 1.

To generate high-gain beams, the mode order of multiple OAM beams used for superposition must be continuous, and the mode spacing should be 1. After determining the values, the antenna patterns of single-mode OAM waves, MG {1,2,3,4,5}, MG {1,2,3,4,5,6,7,8,9,10}, and MG {1,2,...,30} can be obtained. It can be seen that the more array elements used for superposition in the mode group, the higher the beam gain and the narrower the 3 dB beam width. Using the basic correlation method, antenna patterns with different modal numbers can be obtained.

**Figure 2:**
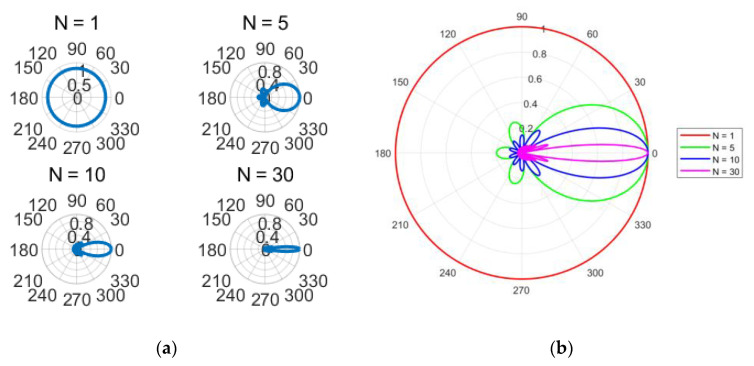
Antenna patterns of different modal numerical groups. (**a**) Antenna patterns with different modal numbers; (**b**) Comparison of antenna patterns with different modal numbers.

**Figure 3:**
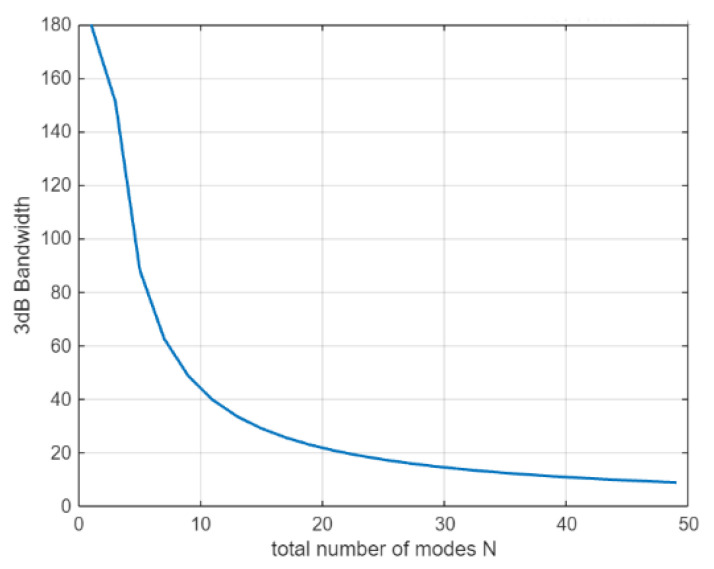
Relationship between 3 dB bandwidth and mode number of the antenna.

Figure 2 above clearly shows that as the number of modes increases, the 3 dB bandwidth of the beam gradually decreases, and the energy distribution becomes more concentrated. Figure 3 further illustrates the relationship between the modal number and the 3 dB bandwidth.

### 3.2. Aggregation Vortex Beam Scanning Method

To achieve a wider range of beam scanning, it is necessary to adjust the initial phase of each mode in the mode group. If the beam is to be directed at a specific angle φ*_d_*, the initial phase of each mode in the mode group must satisfy the following:

(10) $$\varphi_{n}=l_{n}\cdot \varphi_{d}$$

**Figure 4:**
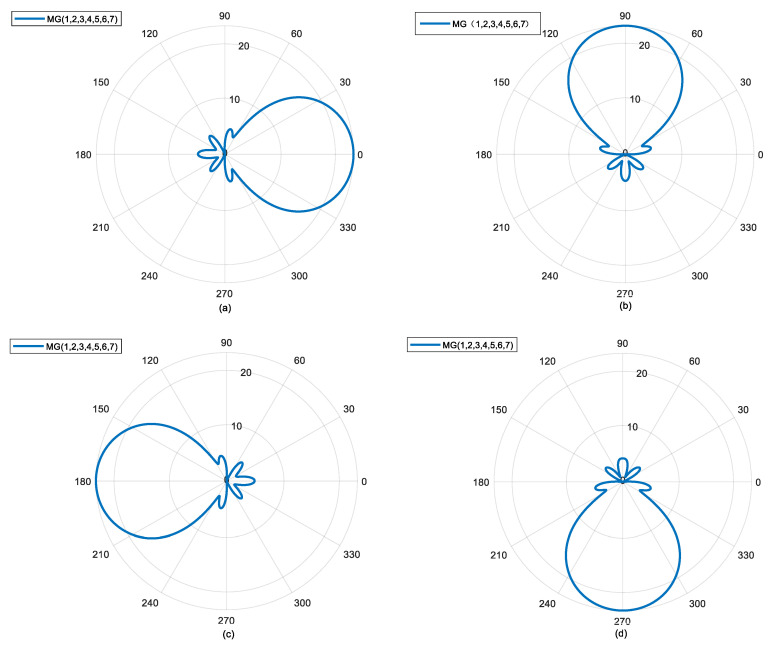
360° undistorted scanning capability of the mode group in azimuth direction; (**a**) azimuth angle of 0°; (**b**) azimuth angle of 90°; (**c**) azimuth angle of 180°; (**d**) azimuth angle of 270°.

The following shows the beam scanning of MG {1, 2, 3, 4, 5, 6, 7} in the antenna angle direction. In Figure 4a, φ*_d_* = 0°, in Figure 4b, φ*_d_* = 90°, in Figure 4c, φ*_d_* = 180°, and in Figure 4d, φ*_d_* = 270°. As an example to further illustrate Figure 4b, in order to make the mode group point in the direction of φ*_d_* = 90°, the initial phase of OAM mode 1 in the mode group should be set to 90°, the initial phase of OAM mode 2 should be set to 180°, and so on. In Figure 4, it can be seen that with a simple phase shift operation, the mode group has the ability to scan 360° in the angle direction, and the beam pattern remains unchanged during the scanning process. In summary, the beam-forming method based on the OAM mode group can achieve omnidirectional distortion-free scanning in the azimuth direction. Compared with the limited scanning angle and complex feeding network of traditional phased arrays, this method has significant advantages.

The four prominent advantages of aggregating the OAM mode group beams are as follows:

1. By adjusting the characteristic parameters of the mode group, the beam shape of the synthesized beam in the angle direction can be flexibly controlled, enabling the generation of highly directional “pencil shaped” beams and multiple beams;
2. The beam constructed by the mode group inherits the vortex phase distribution of traditional OAM beams; that is, it still has vorticity within the main lobe;
3. Due to the vorticity of the mode group and the concentration of energy in the main lobe region, there is quasi-orthogonality between different mode groups;
4. Compared to traditional phased array technology, 360° distortion-free scanning in the azimuth direction can be achieved through simple phase-shifting operations, and the complexity of the feeding structure can be controlled at a smaller level.

## 4. Field and Simulation Verification Design

### 4.1. Simulation Experiment of Aggregated Vortex Beam Generation

#### 4.1.1. Simulation Parameter Design

The simulation parameters are selected as MG {1, 2, 3, 4, 5, 6, 7}, with a total of 14 modes considering the positive and negative modes. The angle of OAM should be consistent and oriented at 9°, with a radius of approximately 25 cm for the entire array surface. The configuration parameters of the array are shown in Table 1.

**Figure 5:**
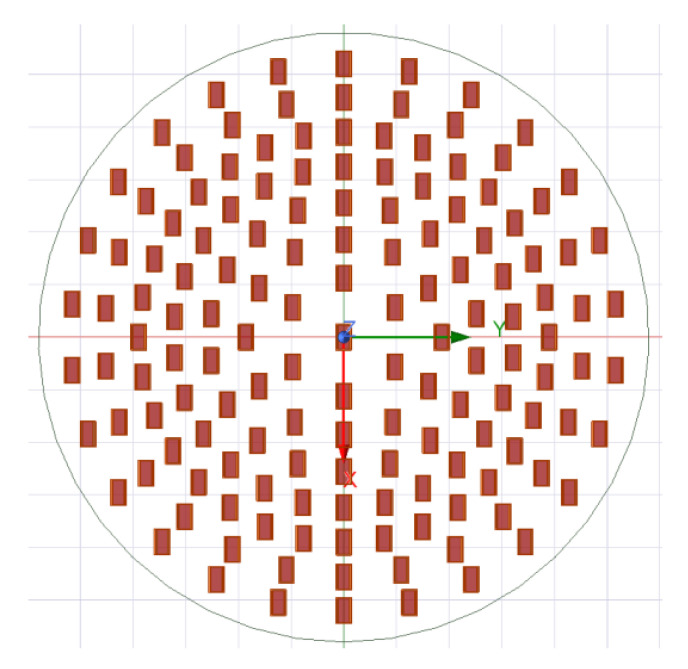
Electromagnetic vortex wave antenna array arrangement.

According to the parameters in Table 1, the array arrangement of the vortex antenna is shown in Figure 5.

**Figure 6:**
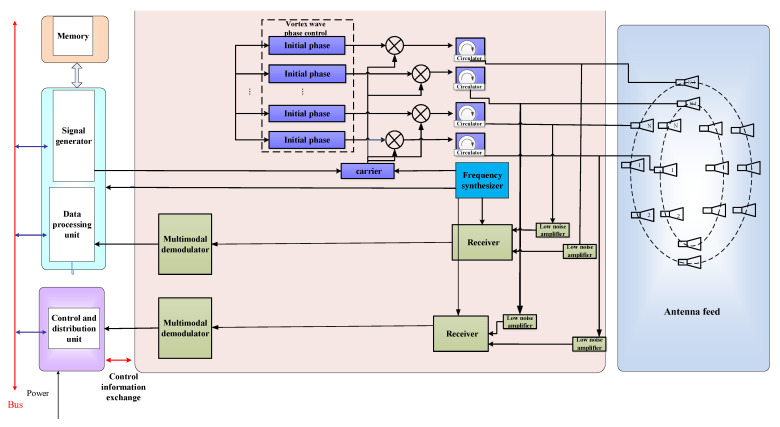
Schematic diagram of antenna feeding structure and electronic equipment used to generate vortex waves.

The antenna adopts a circular array antenna, which has a total of seven loops. The number of the antenna feeding unit from inside to outside is 6, 12, 18, 26, 32, 26, 26. Each antenna circle generates a modal electromagnetic vortex wave through phase control. The connection diagram of the electromagnetic vortex radar equipment is shown in Figure 6.

Each circular antenna corresponds to a feeding port, which feeds the radiating elements on the circular ring with equal amplitude through a power divider network. Then, a phase shifter provides the required initial phase for each radiating element. This is the process of generating electromagnetic vortex waves. For reception, a pulse-based operation is used for transmitting and receiving through a shared antenna. Subsequently, the received signal is decoupled based on the orthogonality of multi-modal information.

#### 4.1.2. Beam Scanning Situation of Different Mode Superposition Mode Groups (With the Same Initial Phase φ=0°)

Four modes are superimposed to form a mode group {l = 1, 2, 3, 4}.

Five modes are superimposed to form a mode group {l = 1, 2, 3, 4, 5}.

Six modes are superimposed to form a mode group {l = 1, 2, 3, 4, 5, 6}.

Seven modes are superimposed to form a mode group {l = 1, 2, 3, 4, 5, 6, 7}.

**Figure 7:**
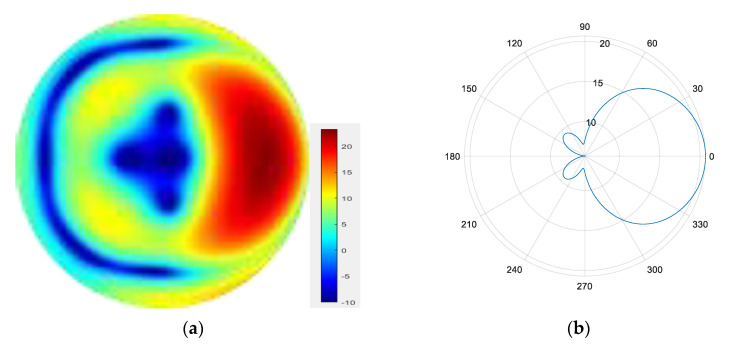
(**a**) Radiation pattern of the four-mode group synthesized beam; (**b**) the directional diagram of the four-mode group in the azimuth direction.

**Figure 8:**
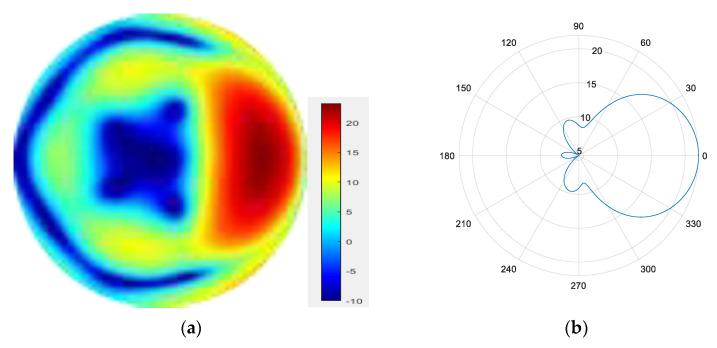
(**a**) Radiation pattern of the five-mode group synthesized beam; (**b**) the directional diagram of the five-mode group in the azimuth direction.

**Figure 9:**
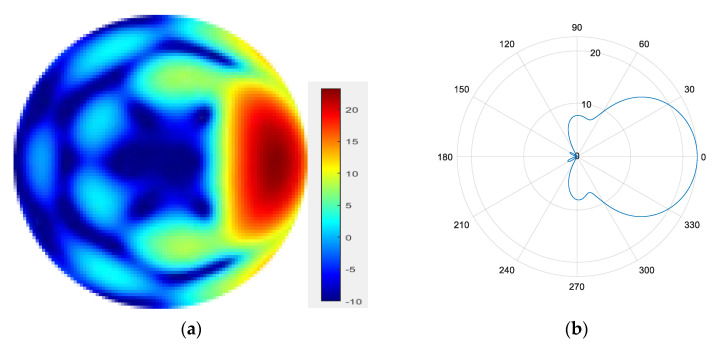
(**a**) Radiation pattern of the six-mode group synthesized beam; (**b**) the directional diagram of the six-mode group in the azimuth direction.

**Figure 10:**
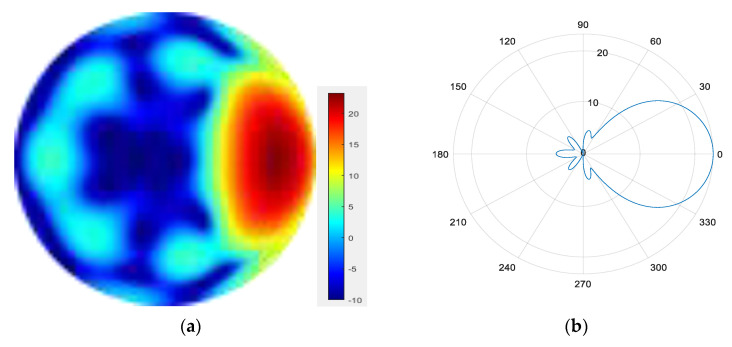
(**a**) Radiation pattern of the seven-mode group synthesized beam; (**b**) the directional diagram of the seven-mode group in the azimuth direction.

From Figure 7, Figure 8, Figure 9 and Figure 10, it can be observed that as the number of modes in the group superposition increases, the back lobe of the synthesized beam becomes smaller and the 3 dB beam width becomes narrower.

#### 4.1.3. Scanning Situation of Mode Group Beams Generated by Superposition of Seven Modes (Initial Phase Changes)

**Figure 11:**
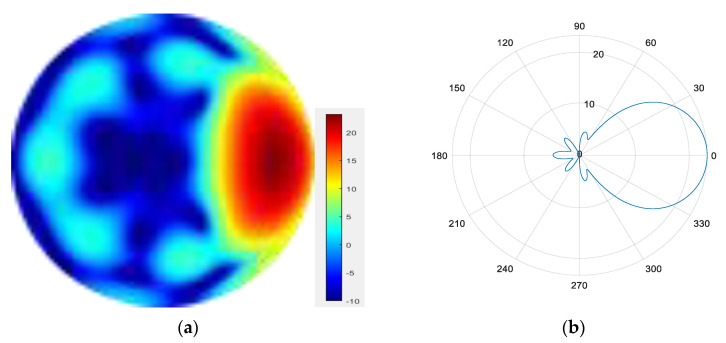
(**a**) φ=0° radiation pattern of synthesized beam; (**b**) φ=0° beam pattern in azimuth direction.

**Figure 12:**
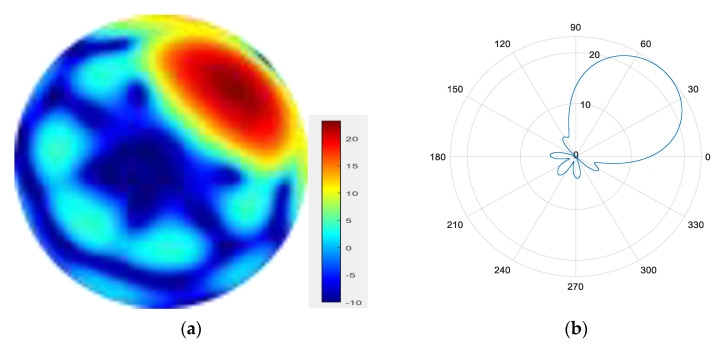
(**a**) φ=45° radiation pattern of synthesized beam; (**b**) φ=45° beam pattern in azimuth direction.

**Figure 13:**
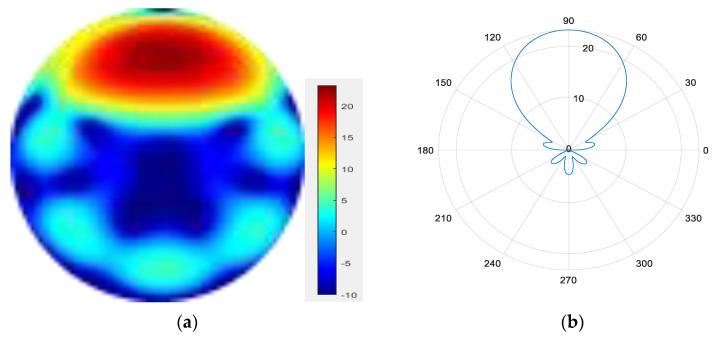
(**a**) φ=90° radiation pattern of synthesized beam; (**b**) φ=90° beam pattern in azimuth direction.

**Figure 14:**
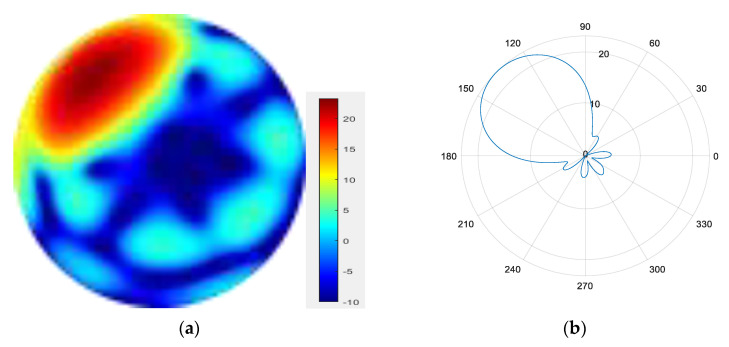
(**a**) φ=135° radiation pattern of synthesized beam; (**b**) φ=135° beam pattern in azimuth direction.

**Figure 15:**
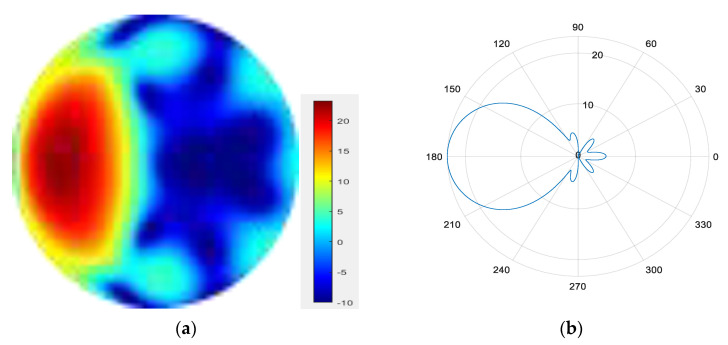
(**a**) φ=180° radiation pattern of synthesized beam; (**b**) φ=180° beam pattern in azimuth direction.

**Figure 16:**
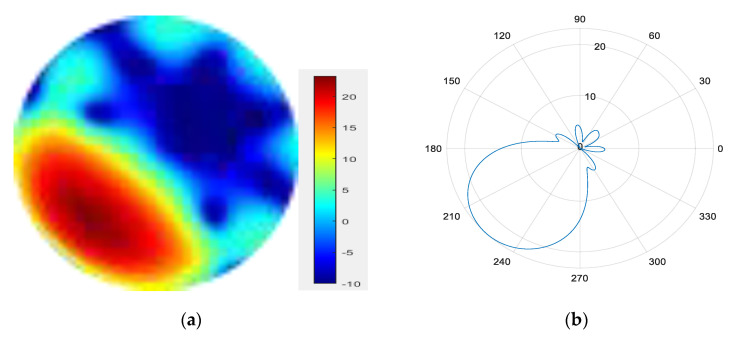
(**a**) φ=225° radiation pattern of synthesized beam; (**b**) φ=225° beam pattern in azimuth direction.

**Figure 17:**
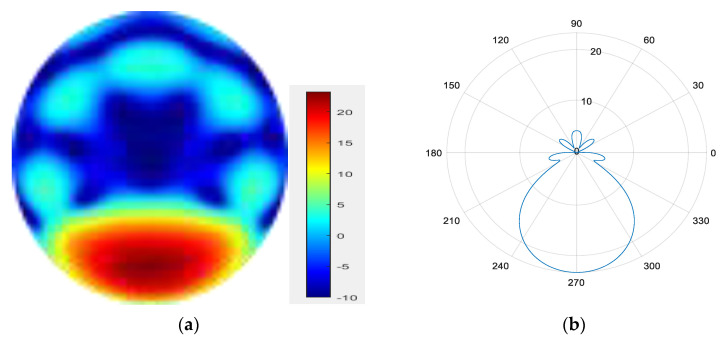
(**a**) φ=270° radiation pattern of synthesized beam; (**b**) φ=270° beam pattern in azimuth direction.

**Figure 18:**
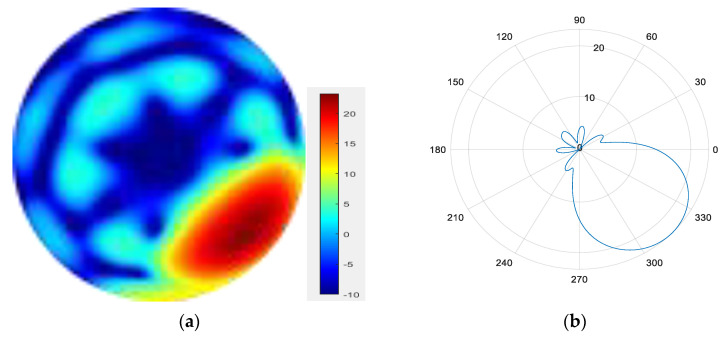
(**a**) φ=315° radiation pattern of synthesized beam; (**b**) φ=315° beam pattern in azimuth direction.

From Figure 11, Figure 12, Figure 13, Figure 14, Figure 15, Figure 16, Figure 17 and Figure 18, it can be seen that changing the initial phase of the aggregated electromagnetic vortex wave can achieve scanning from 0 to 360 degrees, which also indicates that aggregated electromagnetic vortex waves can observe targets from different perspectives.

1.
φ=0°

2.
φ=45°

3.
φ=90°

4.
φ=135°

5.
φ=180°

6.
φ=225°

7.
φ=270°

8.
φ=315°

9. The beams with eight initial phase changes are superimposed together

**Figure 19:**
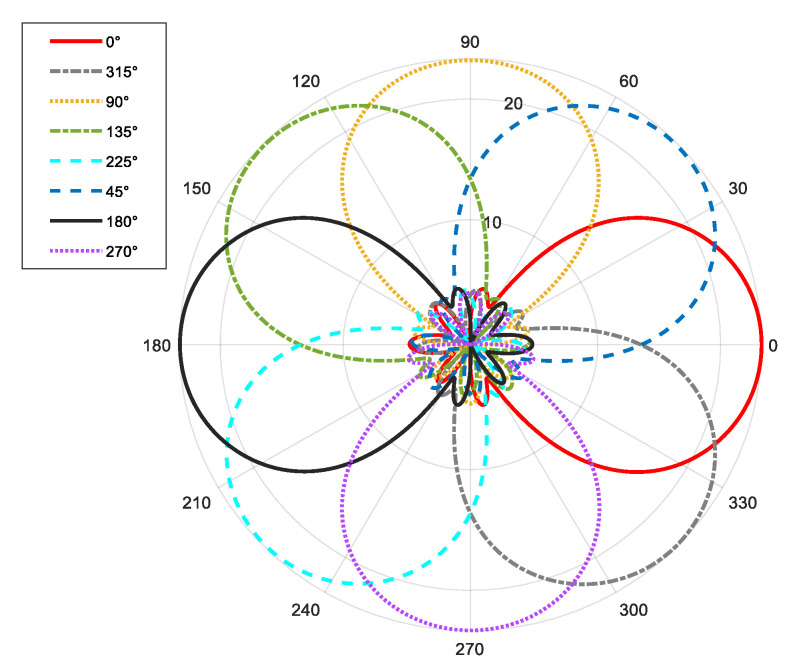
Pattern of eight beam scans driven by initial phase.

From Figure 19, it can be seen that through a simple phase shift operation, the mode group has the ability to scan at an angle of 360°, and the beam pattern remains unchanged during the scanning process. From the perspective of excitation signals, as long as corresponding waveforms are given to the excitation signals of each mode, the aggregation effect of signal power in space can be achieved through direct accumulation.

### 4.2. Field Experimental Results and Analysis

**Figure 20:**
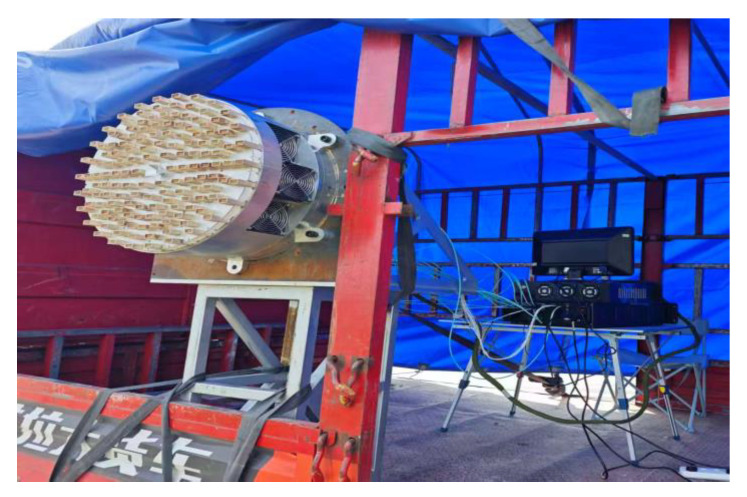
Schematic diagram of vehicle SAR system.

The center frequency of the radar is 10.2 GHz, with a signal bandwidth of 200 MHz and a pulse width of 1 us. The main reason for choosing the X band is that electromagnetic vortex wave radar equipment can be made smaller. In addition, the higher the radar frequency band, the more detailed the targets can be seen. The transmitting power of each array element in each circle is a total of 20 W. The signal is transmitted alternately between pulses in each circle, with a pulse repetition frequency (PRF) of 1 kHz. The distance of the vortex wave reaching the scene is approximately 200 m, and the synthetic aperture time is 1 s. In May 2024, a vehicle-mounted electromagnetic vortex radar imaging experiment was conducted in Chang’an District, Xi’an, China. The schematic diagram of the equipment on the vehicle is shown in the following Figure 20.

The steps of the experiment are as follows:

1. Electromagnetic vortex wave radar electronic equipment emits linear frequency modulation signals;
2. Frequency conversion to X-band through up-converter;
3. Simultaneously transmitting multi-modal information through an electromagnetic vortex wave antenna;
4. The echo signal is received by an electromagnetic vortex wave antenna and sent to electronic devices for data collection;
5. Collecting information and obtaining images through imaging processing.

In the case of vehicle operation, it is also necessary to compensate for the motion and phase errors of the platform [35,36]. Here, the phase gradient autofocus (PGA) algorithm is used, and the process is as follows:

Step 1: Sample selection. The main purpose of this step is to screen high signal-to-noise ratio distance unit sample points. A sufficient number of high signal-to-noise ratio sample points is crucial for the accuracy and efficiency of phase error estimation.

Step 2: Loop shift. First, cyclically shift the azimuth-compressed feature points to the image’s azimuth center and eliminate the linear phase associated with the sample points’ azimuth positions. This preprocessing step facilitates the subsequent phase gradient estimation.

Step 3: Window filtering. Window filtering of sample points can remove most of the clutter and improve the signal-to-noise ratio of the sample points. Here, the window length can be selected through adaptive algorithms. A strategy of gradually reducing the window length can also be pre-set.

Step 4: Phase gradient estimation. After cyclically shifting and windowing the sample points, they are transformed into the azimuth time domain for phase gradient estimation. The phase gradient can be estimated via methods including the linear unbiased least change estimator and maximum likelihood estimator.

Step 5: Iterative phase compensation and estimation. The phase error is obtained by integrating the estimated phase gradient before azimuth compression. Then, the linear component of estimation error is filtered out to prevent image shift. Repeat Steps 2, 3, and 4 until the error estimate converges. If prior information on partial phase errors is obtained, the convergence speed of the PGA can be accelerated.

**Figure 21:**
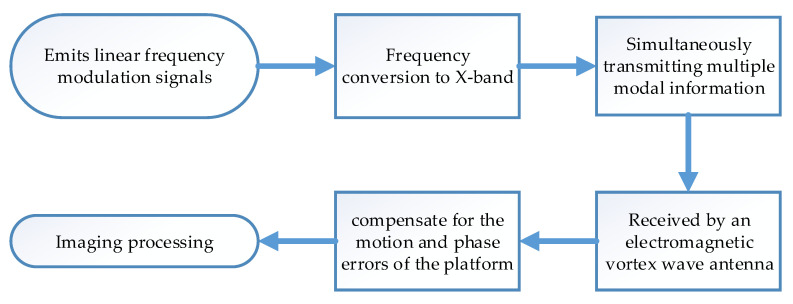
Experimental procedure flow chart.

In order to provide a clearer understanding of the experimental process, we have presented the entire flowchart of the experiment, as shown in Figure 21.

**Figure 22:**
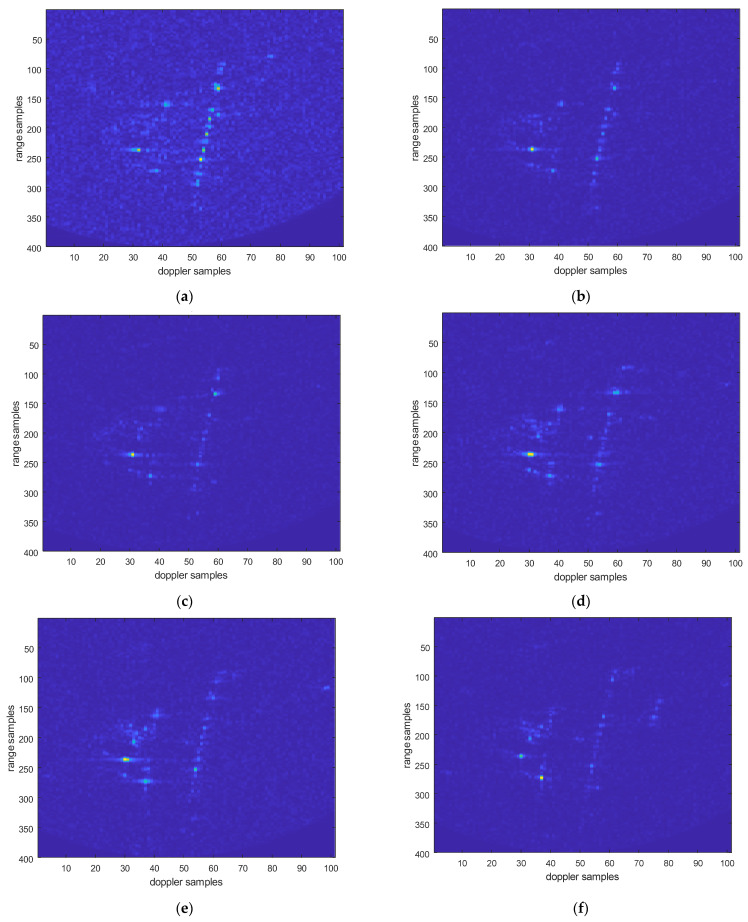

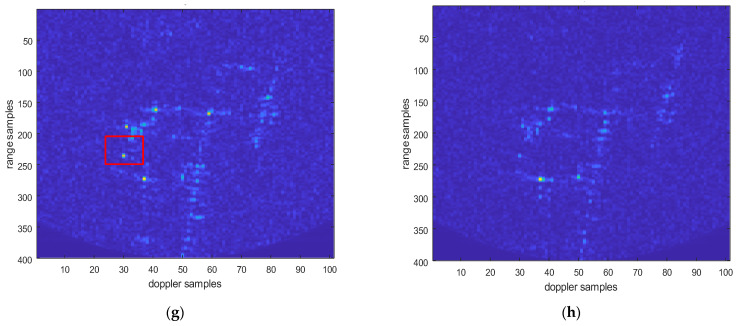
Multi-modal electromagnetic vortex wave SAR image. (**a**) OAM mode MGs1 imaging results; (**b**) OAM mode MGs2 imaging results; (**c**) OAM mode MGs3 imaging results; (**d**) OAM mode MGs4 imaging results; (**e**) OAM mode MGs5 imaging results; (**f**) OAM mode MGs6 imaging results; (**g**) OAM mode MGs7imaging results; (**h**) OAM mode MGs8 imaging results.

An improved chirp scaling algorithm [37,38,39] is used to process data from the vehicle experiments. The following are the processing results of the electromagnetic vortex wave SAR vehicle test data. Figure 22 shows the image of electromagnetic vortex wave SAR after motion compensation and registration, with eight sets of orbital angular momentum (OAM) aggregated vortex wave modal information. Each set of aggregated modal group information contains the energy aggregates of two modes. For example, MGs1 represents modal MG {1, 2}, MGs2 represents modal MG {2, 3}, MGs3 represents modal MG {3, 4}…, and MGs8 represents modal MG {8, 9}.

**Figure 23:**
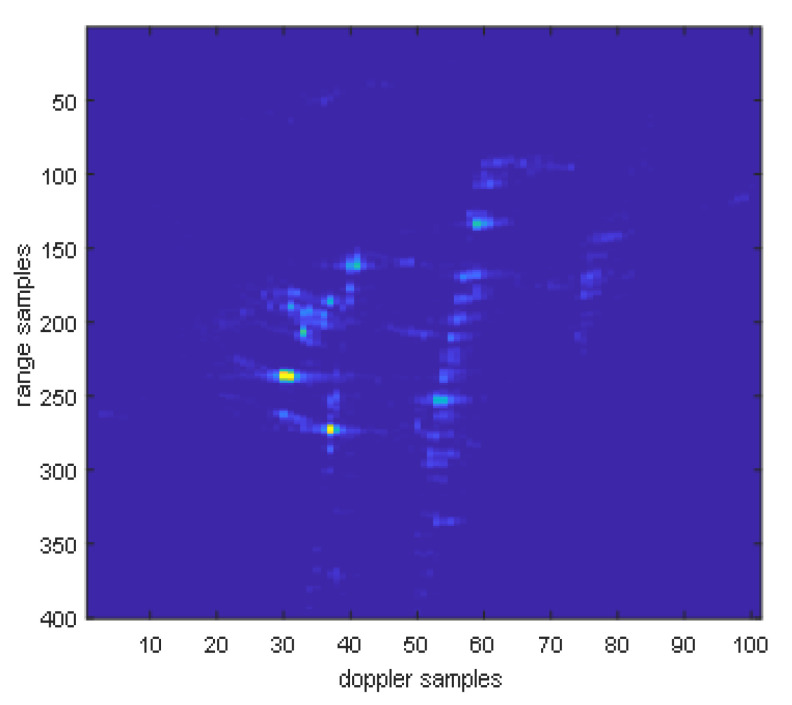
Electromagnetic vortex wave SAR image after fusion.

After fusing multi-modal information, the image is shown in the following Figure 23. It can be seen that the energy of the point target has significantly increased.

**Figure 24:**
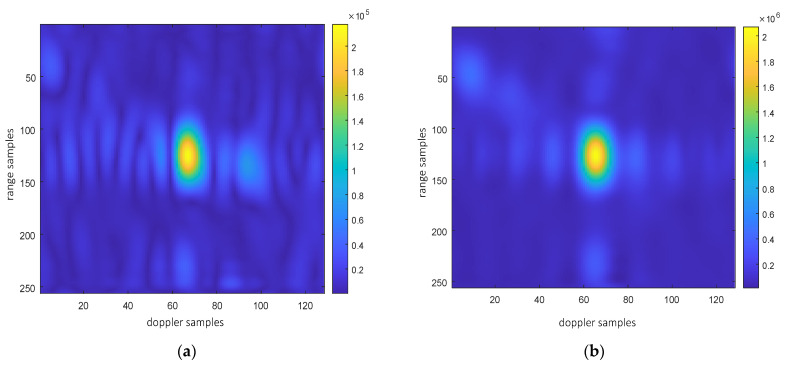
Comparison of point target imaging results before and after non-coherent fusion. (**a**) Before fusion; (**b**) after fusion.

**Figure 25:**
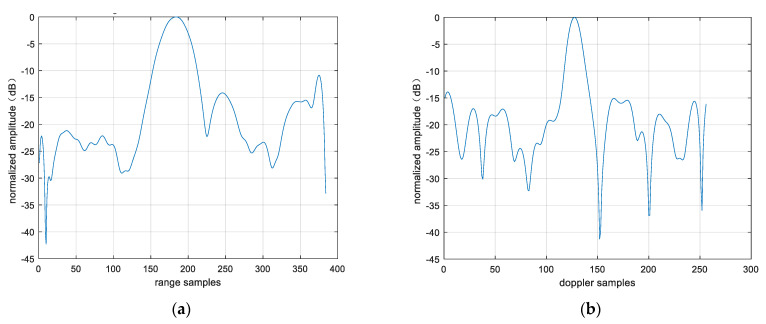
Two-dimensional profile of point target before fusion. (**a**) Rang profile; (**b**) azimuth profile after fusion.

Point targets are extracted for imaging analysis, as shown in the red circles in Figure 23. The extracted point targets are enlarged and processed, as shown in Figure 24. The point targets circled in red in Figure 22g have been selected for comparison. The signal-to-noise ratio (SNR) of the single OAM mode image is 1.2012 dB and the SNR of the fused image is 5.2854 dB. The signal-to-noise ratio after fusion is improved by approximately 4 dB. Then, the 2D profile of the point target before fusion is presented, as shown in Figure 25. The range peak sidelobe ratio is −10.84 dB, and the integrated sidelobe ratio is −10.58dB. The azimuth peak sidelobe ratio is −13.88 dB, and the integrated sidelobe ratio is −7.88 dB.

**Figure 26:**
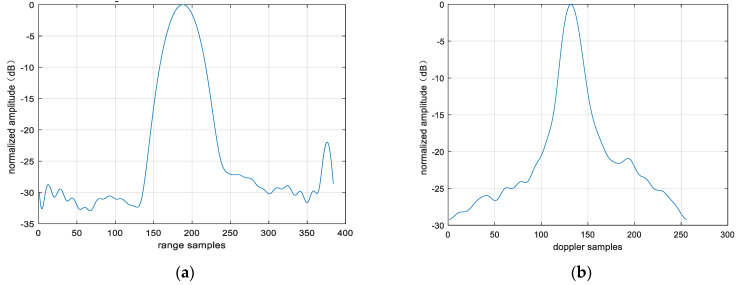
Two-dimensional profile of point target after fusion. (**a**) Rang profile; (**b**) azimuth profile after fusion.

The 2D profile of the point target after fusion is presented, as shown in Figure 26. The range peak sidelobe ratio is −21.95 dB, and the integrated sidelobe ratio is −20.69 dB. The azimuth peak sidelobe ratio is −20.93 dB, and the integrated sidelobe ratio is −15.61 dB. We found that the peak sidelobes and integral sidelobes of point targets were improved after multi-modal fusion. It should be noted that the enhancement of the sidelobes of the point target does not result from fusion itself; instead, the improved sidelobes of the fused point target are attributed to the reduction in image noise. On this basis, the 3 dB width of the point targets in Figure 25 and Figure 26 was analyzed to evaluate the resolution of these point targets. The range resolution before point target fusion is 0.93 m, and the azimuth resolution is 0.35 m. After fusion, the range resolution is 0.96 m, and the azimuth resolution is 0.34 m. The difference in resolution values before and after fusion is not significant, mainly because non-coherent accumulation is used. In addition, the entropy values of the images were also analyzed. The entropy value of the image before fusion was 9.07, and that after fusion was 8.36. The entropy value of the image has decreased significantly, which is also due to the reduction in noise energy after multi-modal fusion.

## 5. Conclusions

This article reports for the first time on the use of multi-modal electromagnetic vortex waves for field experiments. Our research findings demonstrate that, through non-coherent accumulation after the fusion of multi-modal vortex radar images, the energies of both the target’s main lobe and sidelobe signals are enhanced. This enhancement leads to an improvement in the imaging signal-to-noise ratio. Consequently, we can use vortex wave multi-modal information to improve imaging quality. In the future, we will use multi-modal information to improve target resolution, and this part of the work is also being studied.

## Figures and Tables

**Table 1:** Array configuration parameters.

Serial Number	Radius *a* (mm)	Number of Units
#1	56	6
#2	93	12
#3	128	18
#4	162	26
#5	195	32
#6	228	26
#7	260	26

## Data Availability

Dataset available on request from the authors.
